# Implementation of a 3-Tier Priority System for Emergency Department Patients’ Follow-up in Orthopaedic Surgery

**DOI:** 10.5811/westjem.35484

**Published:** 2025-07-13

**Authors:** Samantha M.R. Kling, Christian Rose, Darlene Veruttipong, Sonia Rose Harris, Nadia Safaeinili, Cati G. Brown-Johnson, Sheneé Laurence, Shashank Ravi, Michael J. Gardner, Jonathan G. Shaw

**Affiliations:** * Stanford University School of Medicine, Department of Medicine, Division of Primary Care and Population Health, Evaluation Sciences Unit, Palo Alto, California; † Stanford University School of Medicine, Department of Emergency Medicine, Palo Alto, California; ‡ University of Minnesota, School of Social Work, Saint Paul, Minnesota; § Stanford Medicine, Stanford Health Care, Palo Alto, California; || Stanford University School of Medicine, Department of Orthopaedics, Palo Alto, California

## Abstract

**Introduction:**

Increasing demand for emergency department (ED) services and strained specialty-care access requires referral precision and was the impetus for a collaborative redesign of referrals between the Department of Emergency Medicine and Department of Orthopaedic Surgery.

**Methods:**

Guided by root cause analysis of delays in post-emergency department (ED) specialty follow-up in our academic health system, the intervention targeted the finding that all ED referrals were marked “*urgent*” without differentiation by acuity of orthopedic issues. After implementation, referrals were triaged into three tiers—*immediate*, *urgent*, and *routine—*with stipulated follow-up timeframes. We evaluated differences in completion of scheduling and realized visits, across five calendar months (July–November) pre- and post-implementation (2021 vs 2022). Logistic regression assessed the relationship between patient demographics and outcomes. We report medians and interquartile ranges.

**Results:**

Compared to the 393 *urgent* referrals to the Department of Orthopaedic Surgery pre-implementation, there were 463 total referrals post-implementation as follows: 11/463 (2.4%) marked as *immediate;* 123/463 (26.6%) u*rgent;* and 329/463 (71.1%) r*outine*. Similar proportions successfully scheduled pre- and post-implementation (41.5% vs 45.1%; *P* = .28). On average, *immediate* referrals completed scheduling within 1.0 (0.0 – 1.0) day and were seen in 4.0 (2.0 – 8.0) days, *urgent* referrals completed scheduling within 2.0 (1.0 – 4.0) and 7.0 (5.0 – 15.0) days, and *routine* within 3.0 (1.0 – 6.0) and 12.0 (6.0 – 19.5) days. Race/ethnicity and insurance were related to odds of successful scheduling; Black patients had lower odds than all other groups (odds ratio [OR] 0.3 – 0.4). All insurance categories had higher odds of successful scheduling relative to Medicaid out-of-network (OR 3.5 – 7.2).

**Conclusion:**

A three-tier ED-to-orthopedics referral triage system was quickly adopted and differentiated referrals by urgency but did not impact time to follow-up or loss to follow-up. Structural inequities in access to follow-up care remain.

## THE BOTTOM LINE

Despite rising emergency department (ED) patient volumes and specialty service shortages, timely referrals and care coordination are critical. We evaluated a three-tier system aimed at improving scheduling and follow-up for orthopedic surgery follow-up post-ED visit. Quickly adopted by clinicians, the system prioritized referrals by urgency and facilitated closed-loop communication between ED and schedulers but did not impact overall rates or timeliness of scheduling or follow-ups. It revealed disparities in post-referral access, notably for Black patients and out-of-network Medicaid patients. respectively. This highlights the need to investigate structural barriers to follow-up visit access to inform equity-enhancing, patient-centered solutions.

## INTRODUCTION

### Background and Importance

Recent increases in patient volumes in EDs^1^ and simultaneous shortages in specialty services, including orthopedic surgery, in the US and internationally continue to make referrals and care coordination challenging.^2^–^4^ Difficulty in accessing specialty care after ED visits can lead to repeat ED visits or hospitalizations, further exacerbating record-high hospital volumes and increasing medical costs.^5^ Efforts are needed to improve precision in specialty service utilization through appropriate and timely referrals after ED discharge.

While some musculoskeletal injuries and orthopedic concerns require hospital admission, most can be managed in outpatient settings.^4^ Timely follow-up after ED discharge has been shown to lower likelihood of 30-day revisit and unplanned hospitalizations.^6^–^8^ Efforts to improve coordinated specialty care after ED visits have been broadly attempted, including ED screening and navigator programs, Medicare managed care programs, virtual consults to avoid ED visits,^9^ referral appointment scheduling in the ED, and transitional care nurses.^9^–^17^ Coordination and management programs, however, have had mixed effects.^18^ Many of these interventions target specific populations, require extensive resources to implement and sustain, and may not be generalizable to all settings.

### Goals of This Investigation

Here we describe a collaborative intervention between the Stanford Department of Emergency Medicine and Department of Orthopaedic Surgery to redesign the referral process, which included the following: 1) a three-tier priority system; 2) improved ED referral discharge order/instructions; and 3) closed-loop communication between ED and orthopedic schedulers. The adoption of the referrals and its impact on the intervention on both scheduling and completing a follow-up visit (being seen in clinic by an orthopedic surgeon) after referral from ED were our primary outcomes. As a secondary aim, we explored relationships between patient characteristics and outcomes to identify potential inequities in care.

## METHODS

### Overview

The Department of Emergency Medicine and Department of Orthopaedic Surgery at our academic health center collaborated on a quality improvement (QI) project to redesign ED referrals to maximize time-appropriate scheduling and completion of post-ED visits. This project received non-research determination by Stanford University’s Institutional Review Board (IRB68792).

**Population Health Research Capsule**

What do we already know about this issue?

*Despite rising ED volumes and limited specialty services, such as orthopedic surgery, prompt referrals and care coordination are critical but inconsistent*.

What was the research question?

*Our goal was to evaluate the adoption and impact of a 3-tier system for follow-up referrals from the ED to orthopedic surgery*.

What was the major finding of the study?

*Immediate referrals were scheduled in 1 (0* – *1) day [median (IQR)], urgent within 2 (1* – *4) days, and routine within 3 (1* – *6) days*.

How does this improve population health?

*Our streamlined 3-tier referral system effectively prioritized without overburdening the ED or orthopedic clinics for precise allocation of follow-up care*.

### Institutional Setting

This QI project was a collaboration between the Department of Emergency Medicine and Department of Orthopaedic Surgery of Stanford Medicine (Palo Alto, CA). Stanford Health Care’s ED is a Level I trauma center staffed by 90 full-time attending physicians and 60 resident trainees and conducts over 95,000 patient encounters annually. Stanford Health Care’s Orthopaedics and Sports Medicine service line has five outpatient clinics focused on orthopedic injuries with 60 clinicians conducting 135,000 outpatients encounters annually. Orthopedic surgery is the most referred-to specialty from the Stanford ED with, on average, 64 ED referrals per month to its clinics (including Orthopaedic Surgery, Hand and Spine Surgery, Rehabilitation, and Sports Medicine).

### Intervention Origin

**Figure 1:**
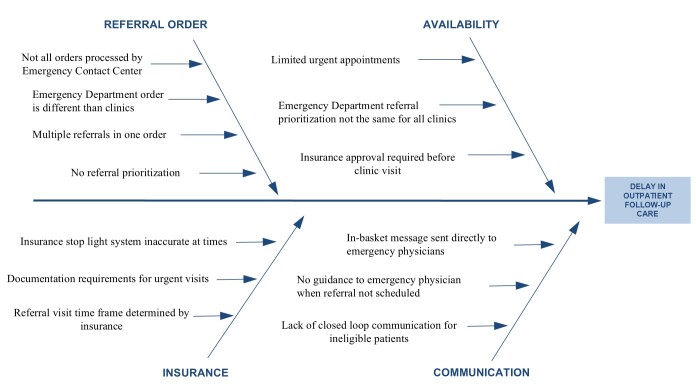
Root cause analysis of emergency department to orthopedic referrals.

In 2021, the ED conducted a root cause analysis based on informal interviews to determine causes of delay in outpatient follow-up after referral from the ED (Figure 1). One identified cause was that all referrals were labeled as *urgent*, without differentiation between high- and low-acuity orthopedic issues or recommended follow-up timing. Thus, schedulers’ work queue could not be organized by urgency of need for follow-up care. Furthermore, all ED referrals were directed internally to the Stanford Department of Orthopaedic Surgery, but the schedulers did not have a consolidated method for determining whether a patient could feasibly follow up with Stanford given their insurance coverage. There was also a lack of available appointment slots in clinics even when a patient was identified as having an acute, time-sensitive need for follow-up. Finally, there was no process for closed-loop communication with patients who were not able to follow up at Stanford. For example, patients who subsequently discovered their insurance would not cover an outpatient visit to Stanford Orthopaedics would not receive communication to offer options for other services.

As part of Stanford’s formal QI system, the Improvement Capability Development Program, the Department of Emergency Medicine aimed to address these root causes in collaboration with the Department of Orthopaedic Surgery.^19^

### Intervention

A comprehensive, multidisciplinary team was convened, which included physicians, nursing, operations personnel, and electronic health record (EHR) information technology staff from both departments along with representatives from the institution’s centralized scheduling center, billing, and a project manager. The team determined that an intervention needed to have the following: 1) clearly displayed priority levels; 2) a specified workflow for each priority designation; and 3) a fallback care plan if patients could not be scheduled within specified timelines.

**Figure 2:**
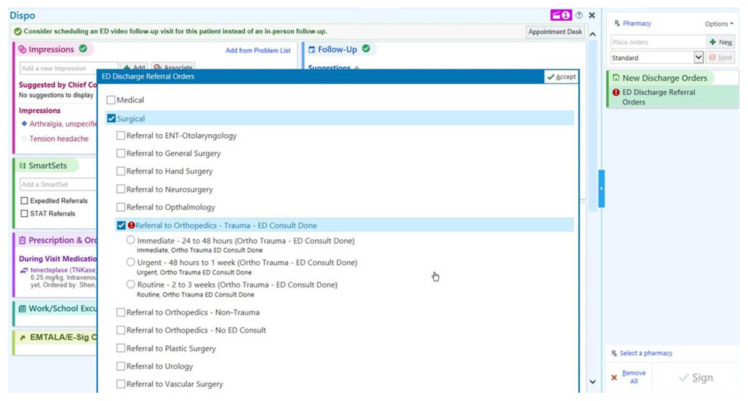
Screenshot of the view of the emergency department-to-the-Department of Orthopaedic Surgery referral priority options within the electronic health record. *ED*, emergency department.

The resulting intervention was a three-tiered referral system based on the urgency of ED-to-Orthopaedic Surgery referrals with recommended follow-up timelines (Figure 2). Referrals could be marked as *routine*, *urgent*, or *immediate* by the emergency physicians based upon the expected time demand of the related injury. *Emergent* follow-up was for those conditions that required orthopedic evaluation and/or management within 48 hours (eg, unstable fractures), *urgent* for those requiring evaluation within one week (eg, fractures reduced and splinted in the ED but requiring casting as an outpatient), and *routine* for those that did not meet the prior criteria and could be seen at a later date (eg, sprains, which may only require conservative management).

All referral orders were sent to the scheduler’s inbox. The scheduler would then prioritize referrals by urgency. If labeled as *immediate*, schedulers attempted expedited insurance review and clearance. If no appointment slot was available in the next 48 hours, they were allowed to overbook a maximum of two patients per week in the Orthopaedic Clinic. If the patient could not make the scheduled appointment or was not able to follow up with Stanford Orthopaedics, the case was sent to the ED callback nurse who would evaluate whether there was another viable follow-up option at a different outpatient location or if the patient would need to return to the ED for further in-patient management. Finally, if there was any question with follow-up plans, the callback nurse shared the case with the emergency telemedicine physician who determined the acuity of follow-up needed based on the injury pattern and prior attempts at scheduling.

The three-tier referral system and workflows for *immediate* referral were implemented June 15, 2022, in the adult ED and its associated observation unit (clinical decision unit). The intervention targeted all referrals to Orthopaedic Surgery. However, musculoskeletal injuries to the hand and spine (1% of referrals to orthopedics) were not included as they have idiosyncratic coverage by rotating services (eg, plastic surgery or hand orthopedic services, and orthopedics or neurosurgery) and, thus, they have unique, institution-specific follow-up plans determined by the on-call service for the day of injury. Similarly, some Orthopaedic Surgery referrals at our institution were sent to the Sports Medicine clinic, which has limited coverage and institution-specific follow-up plans; so they were not included in this more generalized discharge follow-up process. The ED faculty and residents were educated on the new referral-priority categories and how to use them during June/July faculty and resident meetings.

### Design and Data

We used post hoc, EHR data to capture ED-to-Orthopaedic Surgery referrals and timeliness of scheduling and follow-up care during two seasonally matched consecutive periods: 1) pre-implementation (July 16–November 30, 2021); and 2) post-implementation (July 16–November 30, 2022). Outcomes included as successful were completed scheduling and completed clinic visit by an orthopedic surgeon within 90 days of ED discharge; those with completed scheduling or seen in clinic >90 days post-discharge were deemed unlikely related to the index ED encounter.

We also used EHR data to capture patient characteristics, including age, race, ethnicity, preferred language, and insurance coverage type. Patient age at time of the ED encounter and referral was calculated and categorized into three groups: 17–39; 40–64; and ≥65 years of age. We categorized race into the following groups: White, Hispanic, Asian, Black, and other (Native American, Pacific Islander, mixed race, other, and unknown). We used the Hispanic group, the largest non-White racial ethnic group in our study, as the reference group for race and ethnicity in alignment with recent recommendations for quantitative equity research.^20^–^22^ Patient preferred language was grouped into the following categories: English; Spanish; and other language. The patients’ insurance coverage at the ED encounter were categorized into 4 groups: (1) private and military; (2) Medicare; (3) Medicaid out-of-network; and (4) Medicaid in-network, which consisted only of Health Plan San Mateo (HPSM). HPSM is a public Medi-Cal/Medicaid insurance plan for San Mateo County low-income residents. Military was grouped with private insurance due to the limited number of patients (n=7) possessing this insurance and their access to follow-up care was believe to be comparable to patients having private insurance.

### Outcomes

We assessed adoption of the three-tier referral system with two outcomes: 1) number of ED-to-Orthopaedic Surgery referrals placed pre- and post-implementation; and 2) proportion of *routine*, *urgent*, and *immediate* referrals placed post-implementation. The impact of the three-tier referral systems on completed scheduling and being seen in clinic by an orthopedic surgeon was described for the pre- and post-implementation period with two proportional outcomes: 1) referrals with completed scheduling for follow-up; and 2) referrals with patients successfully seen in clinic. Completion of scheduling is dependent on both the health system (ie, schedulers) and patient actions but were considered together here. Timeliness of scheduling and follow-up care was measured with two outcomes: 1) days from referral date to follow-up appointment scheduling date (“completed scheduling” date); and 2) days from referral date to date follow-up encounter was attended (“seen in clinic” date).

### Data Analysis

We calculated descriptive statistics to describe differences in the outcomes across the two time periods. We reported medians and interquartile ranges because data were not normally distributed. Descriptive results are presented as bi-weekly averages pragmatically to address the half month in July and to be amenable to small numbers in some referral categories. Statistical significance was estimated by using chi-square test, the Fisher exact test, and the Monte Carlo estimate of the Fisher exact test, with seed set to one for categorical variables. We determined differences in timeliness between *routine* and *urgent* referrals with the Wilcoxon-Mann-Whitney test as the outcomes were not normally distributed; the *immediate* referral priority was not included due to small numbers. We used univariate logistic regression models to assess the odds of completed scheduling by implementation period.

For secondary analyses, we collapsed data across all patients’ pre- and post-implementation periods as the sample size for some key patient characteristics was small. First, unadjusted logistic regressions with each patient characteristic variable were conducted to assess odds of completing scheduling and odds of being seen in clinic. Finally, we performed multivariable logistic regressions with all patient characteristics. Hispanic was used as the reference group in analyses to de-center Whiteness as the standard experience and avoid “othering.”^20^–^23^ Further, we included all pairwise comparisons in the multivariate model to compare racial and ethnic differences in having scheduling completed. Results of these analyses prompted sensitivity analyses that focused on Black patients; a similar statistical approach was used. *P*-values < .05 were considered statistically significant. Mean (interquartile ranges at 25^th^ and 75^th^ percentiles) are reported. We performed analyses using SAS v9.4 (SAS Institute, Inc., Cary, NC).

### Data Availability Statement

The data that support these findings are available from the corresponding author upon reasonable request.

## RESULTS

### Adoption: Referral Utilization

**Figure 3:**
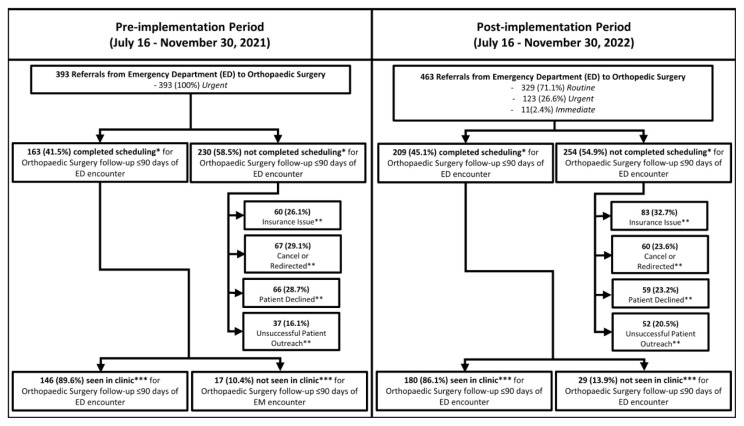
Number (%) of referrals from the emergency department encounter to Orthopaedic Surgery for follow-up, and number (%) of referrals with completed scheduling and follow-up visit attendance. All referrals were marked as *urgent* pre-implementation, whereas referrals could be marked as *routine*, *urgent*, or *immediate* with defined follow-up timelines post-implementation. There were no significant differences in proportion of referrals with completed scheduling (*P = .28; chi square), a recorded reason for referral not being scheduled (**P = .13; Monte Carlo estimate of the Fisher exact test), and follow-up visit attendance (***P = .82; Fisher exact test). *EM*, emergency medicine.

**Figure 4:**
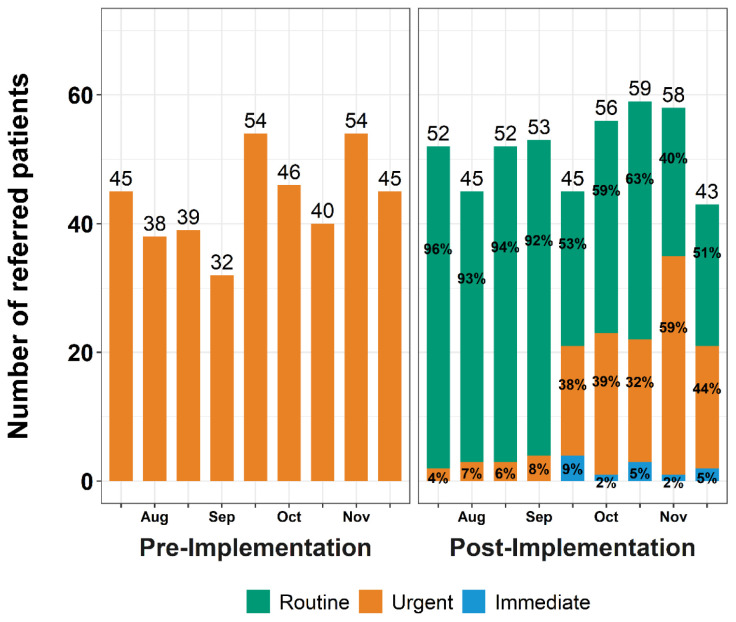
Number of Emergency Department (ED) to Orthopaedic Surgery referrals placed every two weeks (bi-weekly) and emergency department clinicians’ use of referral priorities before (pre) and after (post) implementation of a 3-tiered referral priority system. The number of Emergency Department to Orthopaedic Surgery referrals is provided above each bar. The percentage of referrals that are *Routine*, *Urgent*, and *Immediate* are provided within the green, orange, and blue portion of the bars, respectively.

Figure 3 shows the number of referrals placed in the pre- and post-implementation periods and the priority of referrals to Orthopaedic Surgery (excluding Hand, Spine, and referrals that were redirected to Sports Medicine). In the pre-implementation period, the ED made a total of 393 referrals to Orthopaedic Surgery, 45.0 (IQR 39.0–46.0) referrals bi-weekly (every two weeks), all of which were *urgent* (100%) (Figure 3). Post-intervention, there were a total of 463 ED-to-Orthopaedic Surgery referrals, a median of 52.0 (IQR 45.0–56.0) referrals bi-weekly; 329 referrals (71.1%) were *routine*, 123 (26.6%) were *urgent*, and 11 (2.4%) were *immediate*. Table 1 shows characteristics of referred patients for those who did and did not complete scheduling; results are discussed in the “Scheduling by Patient Characteristics” section below. Figure 4 displays the number of referrals placed and their priority. In the first two months of implementation, emergency clinicians used the *routine* priority for almost all referrals to Orthopaedic Surgery whereas the *urgent* priority was rarely used. Use of the different type of referrals changed in the latter 2.5 months of the post-implementation period; use of the *routine* referral decreased the number of *urgent* referrals, and emergency clinicians used the *immediate* priority sparingly.

### Referral to Completed Scheduling of Follow-up

Approximately the same proportion of referrals were successfully scheduled; 163 of 393 (41.5%) referrals were scheduled in the pre-implementation period, and 209 of 463 (45.1%) in the post-implementation period (*P* = .28 (Figure 3)). Odds of referrals being scheduled did not differ between periods (OR 1.2; 95% confidence interval 0.9–1.5). Scheduler-documented reasons for incomplete scheduling are shown in Figure 3 and did not vary significantly between periods (*P* = .13).

**Figure 5:**
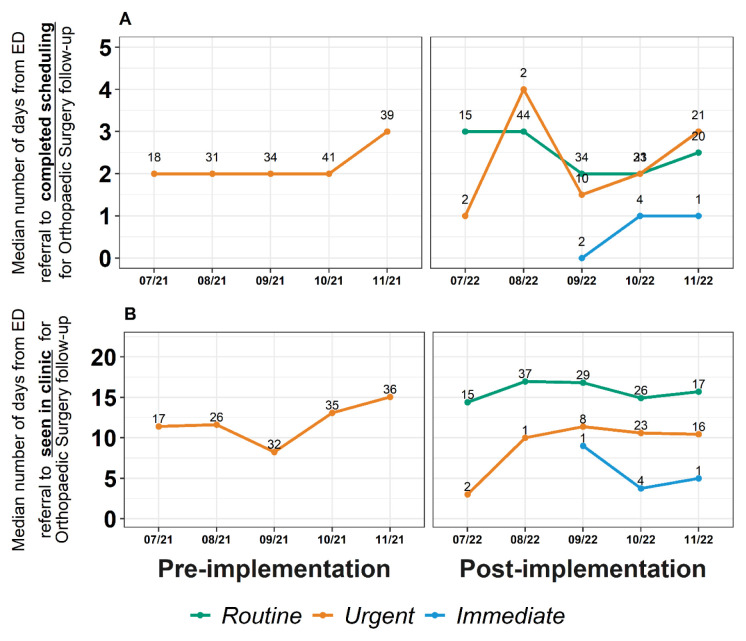
A: Days from referral from the emergency department (ED) to completion of scheduling for Orthopaedic Surgery follow-up encounter pre- and post-implementation of a three-tiered referral priority system. Number of referrals is provided above each data point. B: Days from referral from the ED to seen in clinic for Orthopaedic Surgery follow-up encounter pre- and post-implementation of a three-tiered referral priority system. Number of referrals is provided above each data point. *ED*, emergency department.

Figure 5A shows the time from referral to completed scheduling of follow-up for r*outine*, *urgent*, and *immediate* referrals for the pre- and post-implementation periods. Overall, time from referral to completed scheduling of follow-up with Orthopaedic Surgery did not differ between the two periods; schedulers were able to connect with patients to schedule an appointment within 2.0 (IQR 1.0–4.0) days and 2.0 (IQR 1.0–5.0) days pre- and post-implementation, respectively (*P* = .24) (Supplemental Materials A). Instead, the three-tier referral system, which was designed to categorize patients by urgency of follow-up needs, did show granular differences in scheduling of patients by prioritized category. As described in Figure 5A and Supplemental Materials A, time from referral to scheduling generally followed the expected pattern: *immediate* referrals were successfully processed fastest by schedulers, faster than *urgent* referrals, which were scheduled faster than *routine* referrals. *Urgent* referrals were scheduled significantly faster than *routine* referrals (*P* = .02) (Supplemental Materials B).

### Referral to Being Seen in Clinic

Approximately the same proportion of patients who completed scheduling were seen in clinic; 146 of 163 (89.6%) of scheduled follow-up encounters were seen in clinic during the pre-implementation period, and 180 of 209 (86.1%) were seen in clinic during the post-implementation period (*P* = .82; Figure 3).

Figure 5B shows the time from referral to seen in clinic date for *routine*, *urgent*, and *immediate* referral priorities for the pre- and post-implementation periods. Overall time from referral to date seen in clinic did not differ between the two periods; patients were seen in clinic after referral within 8.0 (IQR 4.0–15.0) days during pre-implementation and 10.0 (IQR 5.0–18.0) days during the post-implementation period (*P* = .09) (Supplemental Materials A). Instead, the three-tier referral system showed granular differences by referral category (Figure 5B), and *urgent* referrals were seen significantly faster than *routine* referrals (7.0 [IQR 5.0– 15.0] vs 12.0 [IQR 6.0–19.5 days]; *P* = .02 (Supplemental Materials B)).

### Scheduling by Patient Characteristics

To identify disparities as areas of potential inequity, we explored differences in scheduling after referral from ED to Orthopaedic Surgery by patient characteristics; this analysis was done across the pre- and post-implementation periods due to the small sample size for some characteristics of interest. In adjusted models (Table 1), every other insurance type was associated with a higher odds of completing scheduling relative to Medicaid out-of-network insurance (Medicaid in-network, OR 3.5; 95% CI 1.9–6.6; Medicare, OR 7.2; 95% CI 3.7–14.3; private and military, OR 4.8; 95% CI 2.7–8.4). Notably, of the patients with Medicaid out-of-network who did not have completed scheduling, 53 of 103 (51.5%) were Hispanic. As shown in Table 2, Black patients had lower odds of having scheduling completed compared to all other race and ethnicity groups, OR 0.3, 95% CI 0.1–0.7 relative to Hispanics, for example.

We hypothesized that insurance or a particular reason for not scheduling might be over-represented in this population; however, Black patients were evenly distributed across insurance types: 14 of 48 (29%) for private and military; 9 of 48 (19%) for Medicare; 10 of 48 (21%) for Medicaid out-of-network; and 14 of 48 (29%) for Medicaid in-network. Even within only private and military insurance, Black patients were less likely to be scheduled in both the unadjusted and adjusted models (both OR 0.1; 95% CI 0.0–0.9 (Supplemental Materials C). In regard to reason for not scheduling, schedulers indicated that patient outreach was unsuccessful in 12 of 48 (25%) Black patients: 8 of 48 (17% had insurance issues; 9 of 48 (19%) declined referral; and 10 of 48 (21%) had an inappropriate referral.

Since 326 of 372 patients (87.6%) who had completed scheduling were seen in clinic, the outcome of not being seen in clinic was too rare and cell sizes of patient characteristics were too small (ie, ≤5 patients) to meaningfully evaluate the relationship between the outcome and patient characteristics.

## DISCUSSION

With the number of ED visits in the US continuing to rise—reaching 131.3 million in 2020—optimizing connections to timely outpatient specialty care is crucial to avoid unnecessary hospitalizations or repeated ED visits.^2^–^4^ At the same time, specialty shortages, including within the Department of Orthopaedic Surgery, are growing, exacerbated by the strains that the COVID-19 pandemic put on our health system.^24^ This QI initiative created more precise communication of needed follow-up timelines from the ED to Orthopaedic Surgery. It also purposefully secured appointment availability for patients needing *immediate* follow-up with closed-loop communication between the ED and schedulers. The system was adopted relatively quickly by clinicians, and outcomes followed expected patterns based on referral priority, while the overall average time to follow-up remained unaffected.

Notably, at initial implementation, emergency clinicians switched from the default of designating all referrals as *urgent* (when presented with three options), essentially designating all as *routine* priority for the first two months after implementation. There was no default option for follow-up; however, over time, and consistent with ongoing faculty and resident education and socialization efforts of the new system, referrals transitioned to a combination of *routine*, *urgent*, and *immediate* priorities indicating gradual transition to a new system. This finding is consistent with other EHR implementations, which often require exploration and experience before full adoption of a new system or workflow.^25^ Notably, this system did not rely on consultation with the specialty services as it was designed to allow the emergency physician to determine priority level based on the injury and practice patterns. If, however, a consultant was contacted by the emergency physician, the priority level could be discussed and agreed upon at that time as necessary.

The three-tier referral system also resulted in expected patterns in the timelines from referral to scheduling and referral to being seen in clinic. *Immediate* referrals were successfully processed fastest by schedulers and seen sooner in clinic, faster than *urgent* referrals that were scheduled and seen faster than *routine* referrals. The median time to scheduling or time to clinic for each referral category, however, did not always align with the timelines prescribed when placing the referrals. This variation in expected vs actual timelines could be due to appointment availability, inability to schedule on the weekends, and patients’ preferred appointment day and time. Further, for *immediate* referrals, orthopedic physicians could have determined that an immediate follow-up timeline was not necessary after reviewing the referral and ED work-up.

Our streamlined three-tier system not only effectively prioritized urgency but did so without overburdening the ED or specialty clinics. Other interventions to improve ED-to-outpatient follow-up have had mixed results and/or require extensive resources.^12^,^16^,^17^,^26^,^27^ Higher tech solutions, like machine-learning models, might seem like a good solution in the current EHR paradigm, but input data can be haphazard and predictions inaccurate, meaning that physicians must retain ultimate responsibility for referral choices.^28^ Our findings demonstrate a simple, sustainable system that successfully triaged patients based on the emergency physician’s perceived urgency without requiring additional staffing or data needs.

As with any such intervention targeting timely access, it is crucial to assess whether it potentially reduces or creates care disparities. Notably, this intervention did not negatively impact the total proportion of patients who scheduled or completed follow-up appointments or the overall median timeliness of these activities. The intervention was designed to better triage patients by medical urgency and, thus, more precisely allocate services where and when needed as opposed to a simple first-come-first-served approach. However, since the intervention did not significantly influence the proportion of patients scheduling or completing follow-up appointments, or the timeliness of these activities, it indicates that not all key drivers were addressed across all patient populations for scheduling, completion, and punctuality of follow-ups. Thus, such efforts to meet the realities of varied patient needs and access to follow-up is a key element of equitable, timely delivery of healthcare resources.^29^

To determine whether ED referrals were having differential impacts on subgroups, we pursued exploratory analyses to better understand the qualities of who did and did not ultimately schedule follow-up orthopedic visits. This analysis revealed disparities; Black patients, although contacted by schedulers, had lower odds of ultimately being scheduled for a visit relative to all other race and ethnicity groupings. Insurance status was studied to determine whether scheduling was driven by type of coverage, and we found that those with out-of-network public insurance—a predominantly Hispanic/Latino population—had lower odds of being scheduled than privately insured patients. Insurance coverage and type of insurance have been shown to impact discharge and transfer rates across EDs, with patients who are uninsured or insured through Medicaid experiencing lower care quality than patients with private insurance.^30^,^31^

Perverse financial incentives influence insurance coverage, hospital payment models, care quality, and policymaking efforts, which may be exacerbated for historically marginalized populations who have been excluded from insurance and healthcare access.^32^ The results of this study highlight similar associations between type of insurance coverage, in particular out-of-network Medicaid, and racial/ethnic identity, respectively, and access to care. As a result, efforts to address inequities in emergency care must simultaneously highlight structural inequities influencing care access and quality, while also focusing care team attention on structural barriers that require culturally sensitive, multifaceted interventions to support equitable care for all patients. In this case, our QI effort aimed to ensure that all patients needing orthopedic referral, regardless of their insurance status, were given the option of a follow-up appointment with Orthopaedic Surgery. Our analysis revealed that patients with out-of-network Medicaid (ie, Medicaid options not contracted with the healthcare system) were largely unable to schedule follow-up. However, our analyses also found that some patients chose not to schedule follow-up appointments. Determining whether this was a result of insurance status vs patient attitudes or experiences that potentially vary by racial or ethnic group toward care options warrants further exploration through qualitative efforts to learn more about the experiences influencing scheduling decisions among patients in this group.

This study of a discrete intervention to improve timeliness of ED-to-Orthopaedic Surgery care transitions demonstrates that while structured referrals improve *precision of* referrals, interpersonal-level QI alone may not be sufficient in promoting health equity. Policy-level and further qualitative work to understand the experiences of patients of color and patients with Medicaid insurance coverage will be crucial to promote equitable care access. Further attention to how similar simple but impactful process changes impact equitable access is crucial, and formal assessments can illuminate disparities and opportunities to redress them.^33^–^35^

## LIMITATIONS

This evaluation was conducted at a single institution, which may limit generalizability. Further, the institution did not have a comparable ED to serve as a control, and thus, we were limited to a pre/post design. The relatively small sample size for *immediate* referrals limited our ability to fully assess the impact on scheduling and follow-up for this sub-group. Also, for *immediate* referrals, we did not have measures of how often appointments were overbooked or how often the case was sent back to the ED callback nurse, two key components of the workflow to enable follow-up care within 24–48 hours. Neither were we able to investigate differences between patients with in-network private insurance and out-of-network private insurance in this retrospective analysis; future work should consider tracking this in real time. Similarly, we were unable to include patient-level factors in our primary analyses due to small sample sizes for characteristics of interest. Furthermore, investigating why patients did not schedule follow-up was limited as documentation of such detail in the EHR is manual and varied across schedulers; however, mistrust of healthcare institutions has been a widely described as a reason for not using healthcare services.^36^,^37^,^38^

## CONCLUSION

This QI initiative demonstrated the feasibility and value of a streamlined three-tier referral system for orthopedic follow-up after ED discharge. The system was readily adopted by clinicians and appropriately stratified patients based on urgency. Notably, it did not impact overall rates of scheduling or time to follow-up but allowed for precise and tailored prioritization and communication of patient needs. Importantly, our analysis revealed disparities in access after referral. Black patients had lower odds of scheduling compared to patients of other races and ethnicities. Further, patients with in-network Medicaid, private, and Medicare coverage, respectively, were more likely to be scheduled than patients with out-of-network Medicaid. Further research should investigate structural barriers to follow-up visit access, and patient-centered solutions to support vulnerable populations in accessing timely and appropriate follow-up care.

## Supplementary Information











## Figures and Tables

**Table 1:** Demographic characteristics of emergency department patients referred to Orthopaedic Surgery, stratified by success in completing scheduling, with logistic regression estimates of odds of completed scheduling.

Demographic Characteristics	Did Not Complete Scheduling n (%)	Completed Scheduling n (%)	*P*-value	Odds of Completed Scheduling	

				Unadjusted Model OR (95% CI)	Adjusted Model OR (95% CI)
All	484 (56.5%)	372 (43.5%)			
Age			<.001^a^		
65+ (reference)	122 (25.2%)	145 (39.0%)		-	-
50–64	115 (23.8%)	78 (21.0%)		0.6 (0.4 – 0.8)	1.0 (0.6 – 1.6)
30–49	153 (31.6%)	83 (22.3%)		0.5 (0.3 – 0.7)	0.9 (0.5 – 1.5)
17–29	94 (19.4%)	66 (17.7%)		0.6 (0.4 – 0.9)	1.0 (0.6 – 1.9)
Race			<.001^a^		
Hispanic (reference)	139 (28.7%)	89 (23.9%)		-	-
White	189 (39.0%)	166 (44.6%)		1.4 (1.0 – 1.9)	0.9 (0.5 – 1.4)
Asian	55 (11.4%)	62 (16.7%)		1.8 (1.1 – 2.8)	1.1 (0.6 – 2.0)
Black	39 (8.1%)	9 (2.4%)		0.4 (0.2 – 0.8)	0.3 (0.1 – 0.7)
Native American, Pacific Islander, mixed race, other	62 (12.8%)	46 (12.4%)		1.2 (0.7 – 1.8)	1.0 (0.6 – 1.8)
Language			.40^a^		
English (reference)	388 (80.2%)	305 (82.0%)			-
Spanish	70 (14.5%)	43 (11.6%)		0.8 (0.5 – 1.2)	1.2 (0.7 – 2.2)
Other	26 (5.4%)	24 (6.5%)		1.2 (0.7 – 2.1)	1.0 (0.5 – 1.9)
Insurance			<.001^a^		
Medicaid out-of-network (reference)	103 (21.3%)	19 (5.1%)		-	-
Medicaid in-network	62 (12.8%)	41 (11.0%)		3.6 (1.9 – 6.7)	3.5 (1.9 – 6.6)
Medicare	105 (21.7%)	143 (38.4%)		7.4 (4.3 – 12.8)	7.2 (3.7 – 14.3)
Private and Military	181 (37.4%)	165 (44.4%)		4.9 (2.9 – 8.4)	4.8 (2.7 – 8.4)
Missing	33 (6.8%)	4 (1.1%)		-	-

a Chi-square test.

*OR*, odds ratio; *CI*, confidence interval.

**Table 2:** Pairwise comparison of odds of completing scheduling by race and ethnicity of emergency department patients referred to Department of Orthopaedic Surgery.

	Hispanic OR (95% CI)	White OR (95% CI)	Asian OR (95% CI)	Black OR (95% CI)	Native American, Pacific Islander, Mixed race, other OR (95% CI)
Hispanic	-	-	-	-	-
White	0.9 (0.5 – 1.4)	-	-	-	-
Asian	1.1 (0.6 – 2.0)	1.3 (0.8 – 2.1)	-	-	-
Black	0.3 (0.1 – 0.7)	0.4 (0.2 – 0.8)	0.3 (0.1– 0.6)	-	0.3 (0.1 – 0.7)
Native American, Pacific Islander, Mixed race, Other	1.0 (0.6 – 1.8)	1.2 (0.7 – 1.9)	0.9 (0.5 – 1.6)	-	-

*OR*, odds ratio; *CI*, confidence interval.
