# Supplementary material for: Implementation of a 3-Tier Priority System for Emergency Department Patients’ Follow-up in Orthopaedic Surgery
Source: West J Emerg Med. 2025 Jul 13;26(4):843–52. doi: 10.5811/westjem.35484 (PMC12342605; doi:10.5811/westjem.35484)
Supplement: Supplementary file 1 [file wjem-26-843-s001.docx]

# Supplemental Materials A.

Comparison of timeliness from referral by ED to completed scheduling and being seen in clinic with Orthopaedic Surgery between pre- and post-implementation periods.

|  | Pre-implementation Period | | Post-implementation Period | | Comparison  (P values) |
| --- | --- | --- | --- | --- | --- |
|  |  |  |  |  | Pre- vs. Post- |
| Number of Referrals from Emergency Department (ED) to Orthopaedic Surgery | 393 | | 463 | |  |
|  | n | Median  (IQ Range) | n | Median  (IQ Range) |  |
| Days from Referral from Emergency Department (ED) Date to…. |  |  |  |  |  |
| Completed scheduling for Orthopaedic Surgery Follow-up date |  |  |  |  |  |
| All referral priorities | 163 | 2 (1.0 - 4.0) | 209 | 2.0 (1.0 - 5.0) | 0.24^a^ |
| *Routine* |  | ^b^ | 144 | 3.0 (1.0 - 6.0) |  |
| *Urgent* |  | ^b^ | 58 | 2.0 (1.0 - 4.0) |  |
| *Immediate* |  | ^b^ | 7 | 1.0 (0.0 - 1.0) |  |
| Seen in clinic by Orthopaedic Surgeon date |  |  |  |  |  |
| All referral priorities | 146 | 8 (4.0 - 15.0) | 180 | 10.0 (5.0 - 18.0) | 0.09^a^ |
| *Routine* |  | ^b^ | 124 | 12.0 (6.0 - 19.5) |  |
| *Urgent* |  | ^b^ | 50 | 7.0 (5.0 - 15.0) |  |
| *Immediate* |  | ^b^ | 6 | 4.0 (2.0 - 8.0) |  |

^a^ Wilcoxon-Mann-Whitney test

^b^ Means not calculated as 393 referrals were urgent during the pre-implementation period.

# Supplemental Materials B.

Comparison of timeliness from referral from Emergency Department (ED) to scheduling completion and follow-up clinic appointment with Orthopaedic Surgery between routine and urgent referrals in the post-implementation period.

|  | **Routine** | **Urgent** | **P-value** |
| --- | --- | --- | --- |
| **Days from Referral Date to….** |  |  |  |
| **Follow-up Scheduled (Completed Scheduling Date)** |  |  | 0.02 ^a^ |
| **n (row %)** | 144 (71.3%) | 58 (28.7%) |  |
| **Median (Interquartile Range)** | 3.0 (1.0 - 6.0) | 2.0 (1.0 - 4.0) |  |
| **Follow-up Attended (Seen in Clinic Date)** |  |  | 0.02 ^a^ |
| **N (row %)** | 124 (71.3%) | 50 (28.7%) |  |
| **Median (Interquartile Range)** | 12 (6.0 - 19.5) | 7 (5.0 - 15.0) |  |

^a^ Wilcoxon-Mann-Whitney test was utilized as data was not normally distributed.

# Supplemental Materials C.

Demographic characteristics stratified by Emergency Department (ED) patients who completed scheduling for an Orthopaedic Surgery follow-up visit compared to those that did not complete scheduling among patients who had private or military insurance. Results of logistic regression models assessing demographic characteristics associated with completed scheduling.

| **Demographic Characteristics** | **Did Not Complete Scheduling** | **Completed Scheduling** | **P-value** | **Unadjusted**  **Model** | **Adjusted**  **Model** |
| --- | --- | --- | --- | --- | --- |
|  | **n (%)** | **n (%)** |  | **OR (95% CI)** | **OR** (**95% CI**) |
| **All** | 181 (53.2%) | 165 (47.7%) |  |  |  |
| **Age** |  |  |  |  |  |
| 65+ (ref) | 19 (10.5%) | 12 (7.3%) | 0.25^a^ | - | - |
| 50-64 | 51 (28.2%) | 48 (29.1%) |  | 1.5 (0.7 - 3.4) | 1.6 (0.7 - 3.7) |
| 30 - 49 | 68 (37.6%) | 52 (31.5%) |  | 1.2 (0.5 - 2.7) | 1.3 (0.6 - 2.9) |
| 17-29 | 43 (23.8%) | 53 (32.1%) |  | 2.0 (0.9 - 4.5) | 2.1 (0.9 - 5.0) |
| **Race** |  |  | 0.07^b^ |  |  |
| Hispanic (ref) | 23 (12.7%) | 27 (16.4%) |  | - | - |
| White | 88 (48.6%) | 73 (44.2%) |  | 0.7 (0.4 - 1.3) | 0.8 (0.4 - 1.5) |
| Asian | 33 (18.2%) | 35 (21.2%) |  | 0.9 (0.4 - 1.9) | 0.9 (0.4 - 2.0) |
| African American/Black | 12 (6.6%) | 2 (1.2%) |  | **0.1 (0.0 - 0.7)** | **0.1 (0.0 - 0.7)** |
| Native American, Pacific Islander, Mixed Race, Other | 25 (13.8%) | 28 (17.0%) |  | 1.0 (0.4 - 2.1) | 1.0 (0.5 - 2.4) |
| **Language** |  |  | 0.92^b^ |  |  |
| English (ref) | 174 (96.1%) | 157 (95.2%) |  | - | - |
| Spanish | 4 (2.2% ) | 5 (3.0%) |  | 1.4 (0.4 - 5.2) | 1.3 (0.3 - 5.7) |
| Other | 3 (1.7%) | 3 (1.8%) |  | 1.1 (0.2 - 5.6) | 1.0 (0.2 - 5.4) |

^a^ Chi Square Test

^b^ Fisher’s Exact Test
