# Supplementary material for: Implementation of a 3-Tier Priority System for Emergency Department Patients’ Follow-up in Orthopaedic Surgery
Source: West J Emerg Med. 2025 Jul 13;26(4):843–52. doi: 10.5811/westjem.35484 (PMC12342605; doi:10.5811/westjem.35484)
Supplement: Supplementary file 2 [file wjem-26-843-g002.pdf]

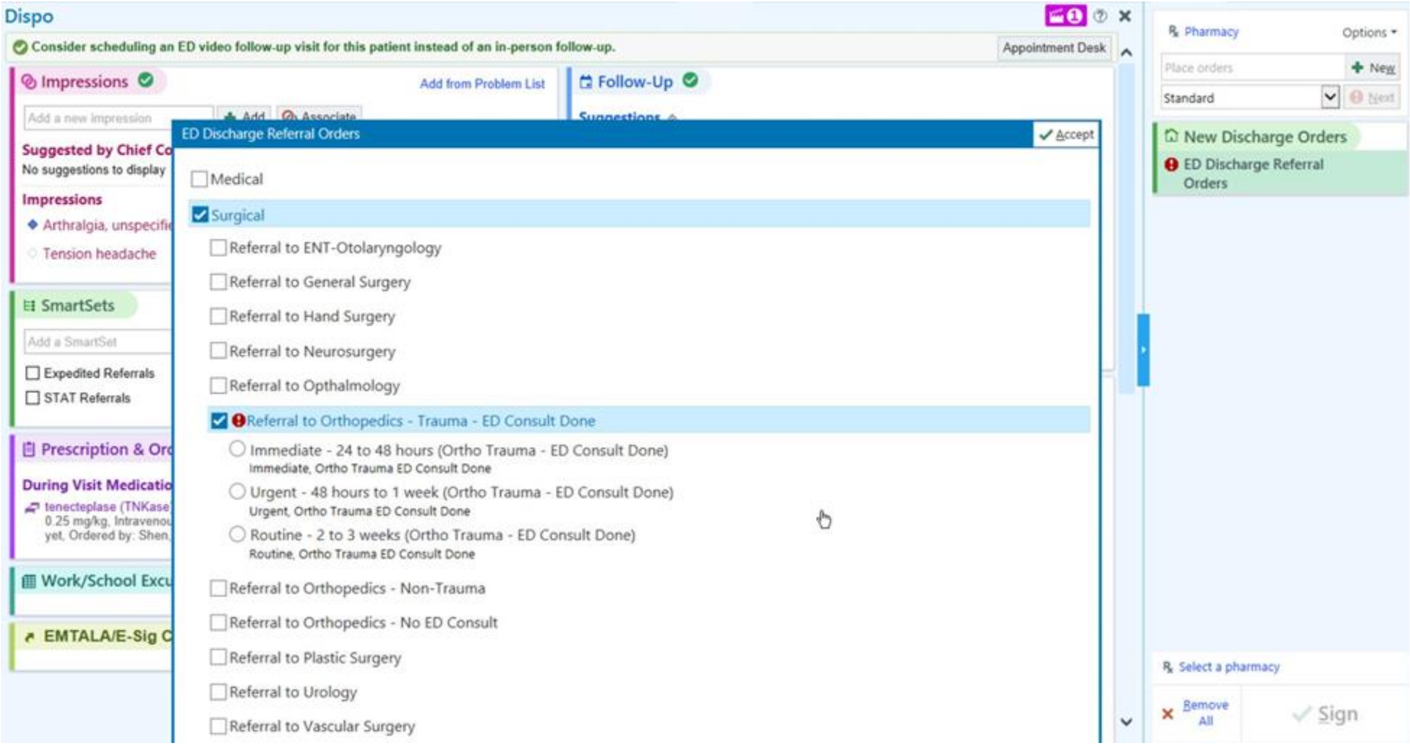
Dispo
Consider scheduling an ED video follow-up visit for this patient instead of an in-person follow-up.
Appointment Desk
Impressions
Add from Problem List
Follow-Up
Add a new impression
Suggested by Chief Co
No suggestions to display
Impressions
Arthralgia, unspecifi
Tension headache
SmartSets
Add a SmartSet
Expedited Referrals
STAT Referrals
Prescription & Ord
During Visit Medicatio
tenecteplase (TNKase
0.25 mg/kg, Intravenou
yet, Ordered by: Shen,
Work/School Excu
EMTALA/E-Sig C
ED Discharge Referral Orders
Accept
Medical
Surgical
Referral to ENT-Otolaryngology
Referral to General Surgery
Referral to Hand Surgery
Referral to Neurosurgery
Referral to Opthalmology
Referral to Orthopedics - Trauma - ED Consult Done
Immediate - 24 to 48 hours (Ortho Trauma - ED Consult Done)
Immediate, Ortho Trauma ED Consult Done
Urgent - 48 hours to 1 week (Ortho Trauma - ED Consult Done)
Urgent, Ortho Trauma ED Consult Done
Routine - 2 to 3 weeks (Ortho Trauma - ED Consult Done)
Routine, Ortho Trauma ED Consult Done
Referral to Orthopedics - Non-Trauma
Referral to Orthopedics - No ED Consult
Referral to Plastic Surgery
Referral to Urology
Referral to Vascular Surgery
Pharmacy
Options
Place orders
New
Standard
Next
New Discharge Orders
ED Discharge Referral Orders
Select a pharmacy
Remove All
Sign
