# Supplementary figures and images for: Implementation of a 3-Tier Priority System for Emergency Department Patients’ Follow-up in Orthopaedic Surgery
Source: West J Emerg Med. 2025 Jul 13;26(4):843–52. doi: 10.5811/westjem.35484 (PMC12342605; doi:10.5811/westjem.35484)

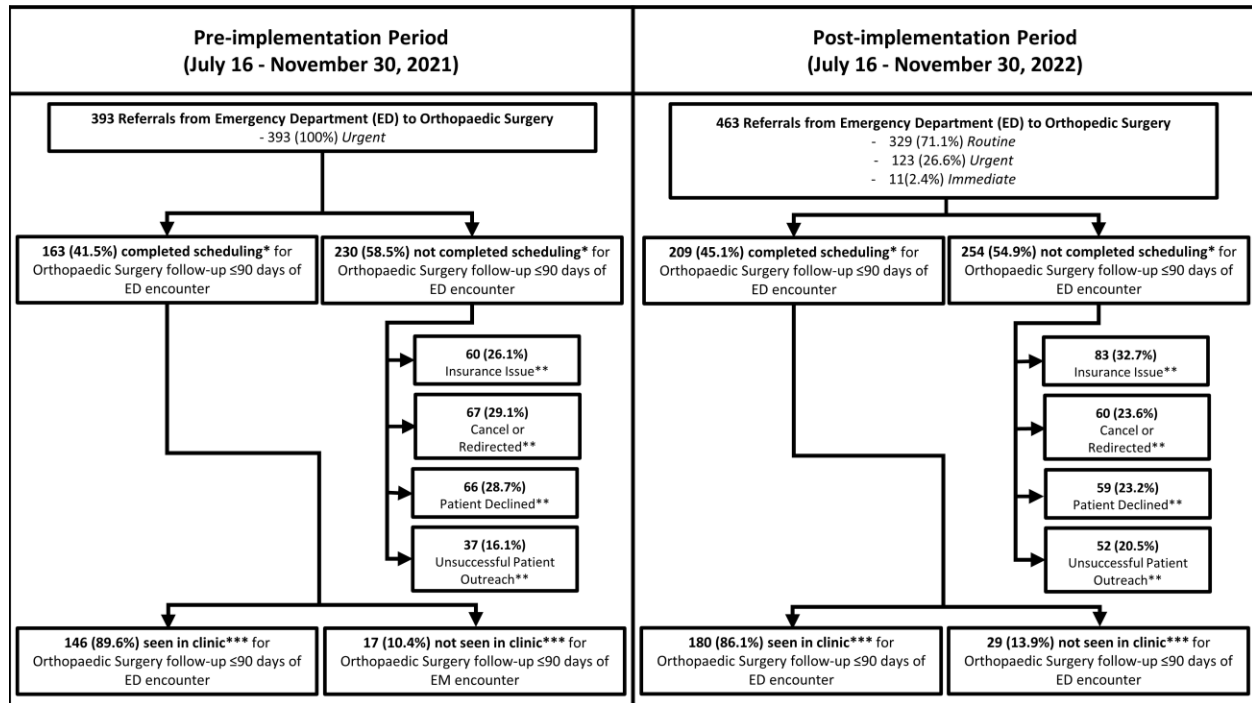

Supplement: Supplementary file 3 [file wjem-26-843-g003.pdf]

Number of referred patients

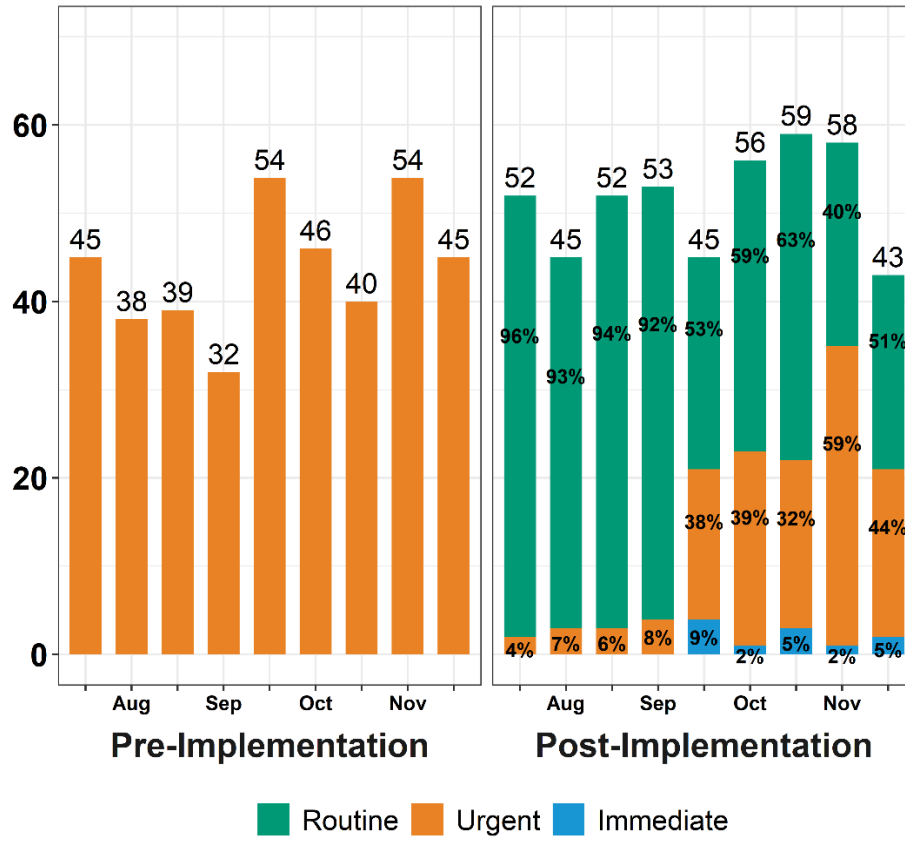

Supplement: Supplementary file 4 [file wjem-26-843-g004.pdf]
