# Supplementary material for: Implementation of a 3-Tier Priority System for Emergency Department Patients’ Follow-up in Orthopaedic Surgery
Source: West J Emerg Med. 2025 Jul 13;26(4):843–52. doi: 10.5811/westjem.35484 (PMC12342605; doi:10.5811/westjem.35484)
Supplement: Supplementary file 5 [file wjem-26-843-g005.pdf]

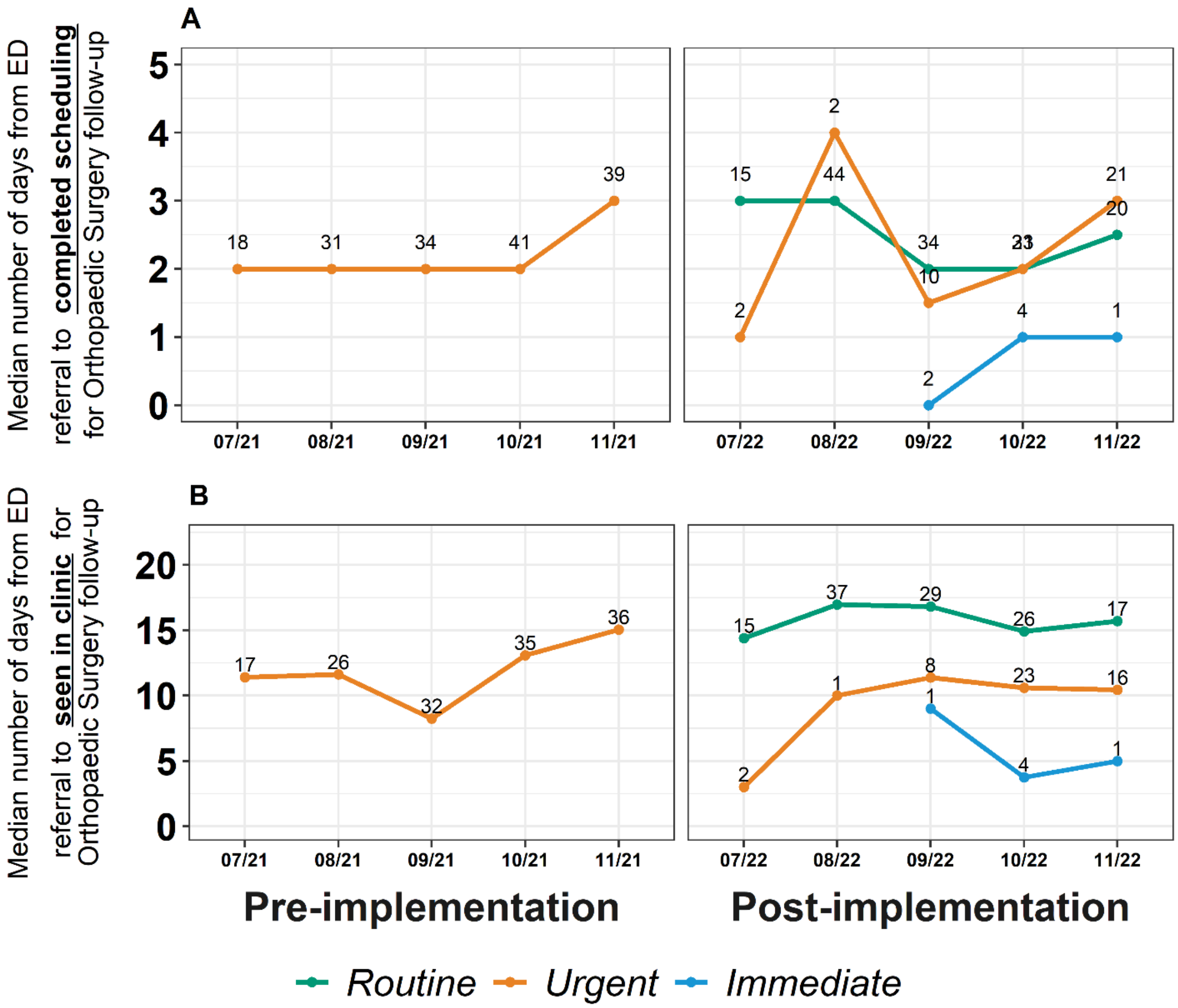
A
B
Median number of days from ED referral to completed scheduling for Orthopaedic Surgery follow-up
Median number of days from ED referral to seen in clinic for Orthopaedic Surgery follow-up
07/21
08/21
09/21
10/21
11/21
07/22
08/22
09/22
10/22
11/22
Pre-implementation
Post-implementation
Routine
Urgent
Immediate
